# Risk Factors Associated with Low Back Pain among A Group of 1510 Pregnant Women

**DOI:** 10.3390/jpm10020051

**Published:** 2020-06-15

**Authors:** Aleksandra Bryndal, Marian Majchrzycki, Agnieszka Grochulska, Sebastian Glowinski, Agnieszka Seremak-Mrozikiewicz

**Affiliations:** 1 Department of Physiotherapy, Institute of Health Sciences, Pomeranian University, 76-200 Slupsk, Poland; agnieszka.grochulska@apsl.edu.pl; 2 Division of Perinatology and Women’s Diseases, Poznan University of Medical Sciences, 61-701 Poznan, Poland; marian.majchrzycki@gmail.com (M.M.); agnieszka.seremek@wp.pl (A.S.-M.); 3 Laboratory of Molecular Biology in Division of Perinatology and Women’s Diseases, Poznan University of Medical Sciences, 61-701 Poznan, Poland; 4 Division of Mechatronics and Automatics, Faculty of Mechanical Engineering, Koszalin University of Technology, 75-453 Koszalin, Poland; sebastian.glowinski@tu.koszalin.pl

**Keywords:** pregnancy, low back pain, VAS, Roland Morris Disability Questionnaire, Oswestry Disability Index

## Abstract

Background: Low Back Pain (LBP) is a frequent, very common, and costly health problem. LBP, which occurs during pregnancy, may become a lifelong problem. The aim of this study was to determine the risk factors associated with LBP in pregnant women. Methods: The study included 1510 pregnant women. A questionnaire assessing demography, lifestyle, prevalence, and characteristics was designed and used in the study. Pain intensity was assessed with the VAS (Visual Analogue Scale). The RMDQ (Roland Morris Disability Questionnaire) was used to assess the effect that low back pain had on the functional capacity of a pregnant woman. Middle (thoracic) and low back pain disability was measured with the help of the ODI (Oswestry Disability Index) questionnaire. Results: The study confirmed that lying/sleeping (49.6%) and sitting positions (38.7%) as well as walking (37.2%) are the most significant factors causing LBP. It was also found that women who had not engaged in physical activity were more likely to experience LBP. Conclusions: Predisposing factors for LBP in pregnancy are LBP in previous pregnancies, back pain during menstruation, a younger age and a lack of physical activity. Most women in pregnancy with LBP experienced minimal and mild disability.

## 1. Introduction

Pregnancy has a profound physiological effect on a woman’s body. It does not only affect cardiovascular, hormonal, and urinary systems but also involves musculoskeletal problems, with a particular impact on the axial skeleton. Significant hormonal changes accompanied by an increase in body mass and a change in the size and position of the uterus contribute to the alteration of the centre of gravity and thus apply additional static and dynamic forces affecting the axial skeleton [1,2].

Recommended gestational weight gain ranges between 11 and 16 kg, of which approximately half is gained in the abdomen [3]. As a result, the growing abdomen triggers postural compensations, which frequently culminate in low back pain (LBP)—the most common musculoskeletal complaint during pregnancy [4]—and/or pelvic girdle pain (PGP) [5]. LBP during pregnancy usually begins in the second trimester, at about 22 weeks. The prevalence of PGP and LBP in pregnant women during the first trimester was estimated at 50% [6]; in the second trimester it ranged between 40% and 70% [7]; and for the third trimester, it was between 70% and 80% [8]. Approximately half of women with initially manifesting LBP during pregnancy continue to have pain 1 year after childbirth [6], and 20% are symptomatic 3 years postpartum [9]. The prevalence of LBP during pregnancy ranges from 20% to 90%; most studies report a prevalence greater than 50% [10,11,12,13,14]. The prevalence of LBP initiated during pregnancy is 61% [15]. PGP typically begins by the end of the first trimester, reaching its peak between 24 and 36 weeks of pregnancy; this usually subsides spontaneously within 6 months postpartum; however, in 8% to 10% of women, the pain persists for 1 to 2 years after birth [16,17,18]. The incidence of PGP in pregnancy ranges from 4% to 76%, depending on the definition used, which often includes LBP. When defined as pain located at the level of the posterior iliac crest and the gluteal fold over the anterior and posterior elements of the bony pelvis, the prevalence ranges from 16% to 25% [18]. However, the precise definition of PGP often coincides with that of LBP, which is typically used for all the frequency index estimates in the literature [17].

The purpose of the study was to determine the risk factors associated with LBP in pregnant women, the effect of LBP on functional capacity, and the degree of disability in pregnant women.

## 2. Materials and Methods

The research sample consisted of 1510 pregnant women admitted to the Gynaecology and Obstetrics Clinical Hospital at the Karol Marcinkowski University of Medical Sciences in Poznan. The female patients enrolled in the study were Polish nationals of a Caucasian origin capable of understanding and filling out the questionnaire in the Polish language. All subjects were healthy pregnant women. The study was carried out between April 2014 and March 2017. The inclusion criteria were pregnancy, participation in routine check-ups, and giving consent to participate in the research study. The exclusion criteria were: 1) a medical history of surgical lumbar spine interventions, 2) cancer, 3) spinal deformities (e.g. scoliosis), 4) osteoporosis, 5) multiple sclerosis, 6) pelvic fractures and other pelvic area problems, 7) inflammatory conditions, and 8) a refusal to participate in the study. All the women participating in the study were given verbal or written information on the purpose of research and the possibility to withdraw their informed consent at any time during the study. The study was approved by the Bioethics Committee of the Karol Marcinkowski University of Medical Sciences in Poznan (no. 372/12).

Data for this study were collected through a questionnaire designed in such a way as to avoid the disclosure of personal details. The authors’ questionnaire included questions about age, height, education, occupational category, average weight before pregnancy and gestational weight (when completing the questionnaire), previous pregnancy childbirth type, and week of pregnancy and a question about the number of pregnancies including the current. The body mass index (BMI) was calculated using the following formula: the weight in kilograms was divided by the height expressed in meters squared. The questionnaire also included questions about pain-related complaints: the occurrence of low back pain before pregnancy and in previous pregnancies (if applicable), and the occurrence of back pain during menstruation (before pregnancy). The last part of the questionnaire concerned physical activity before and during pregnancy. The question was whether during the last 7 days activities involving average physical exertion were performed (causing slightly faster breathing and slightly faster heartbeat, e.g., very fast walking or cycling). It is about activities lasting a minimum of 10 minutes, a minimum of 2 times a week.

Pain intensity was assessed by the VAS (Visual Analogue Scale). The evaluation scale was a ruler measuring 10 cm in length upon which a pregnant woman indicated pain intensity with a finger or with a slider indicator, where 0 stands for “no pain” and 10, “worst pain possible” [19]. The impact of low back pain on the quality of life of a pregnant woman was evaluated by the RMDQ (Roland Morris Disability Questionnaire). It consists of 24 statements describing daily activity limitations, to which a patient answers with “yes” or “no”. The subject was asked to complete the self-administered questionnaire (typically 5–10 min) by choosing the sentences most accurately describing how they felt on the day of the survey (a maximum of 24 items could be checked on the list). The total score varied from 0, denoting no disability, to 24, reflecting the highest level of disability. Patients were divided into four groups depending on how they scored: no disability—0–3 points, mild disability—4–10 points, moderate—11–17 points, and severe—18–24 points [20,21]. The degree of disability caused by middle and low back pain was assessed by the ODI (Oswestry Disability Index) questionnaire. It is considered to be the standard measure in the evaluation of the effect back pain has on the patient’s quality of life. This self-administered questionnaire was completed by answering 10 questions concerning pain intensity, lifting, sitting, sleeping, travelling, personal care, standing, social life, and sexual function. In each topic category of the questionnaire, the patient checks one statement most accurately resembling their situation scored from 0—indicating no disability, to 5—maximum disability. The total possible score is 50. The degree of a pregnant woman’s disability is expressed as a percentage (the higher the score, the higher disability). Patients were put into five groups according to the degree of disability expressed as a percentage minimal disability—0–20%, moderate disability—21–40%, severe disability—41–60%, crippling disability—61–80%, and bed-bound patient—81–100%. The ODI questionnaire is a reliable and valid, responsive, condition-specific assessment tool [21]. The questionnaire used in this study had already been translated into Polish, and its reliability and accuracy had been tested and approved [22,23].

LBP was defined as pain in the lumbosacral spine radiating to one or both legs, lasting for a minimum of one day [24]. All pregnant women participating in the study were asked to complete the questionnaires accurately and truthfully.

Statistical analyses were carried out in the R [25] software with the psych package [26] using Student’s t-test, as well as analysis of variance and post hoc tests as indices of the differences between groups. Furthermore, logistic regression and the odds ratio were employed as indices of pain probability occurrence depending on the analysed factors. As descriptive statistics, the mean, median, variance, and maximum and minimum values were used. The effect size was calculated on the basis of the eta 2 index.

## 3. Results

Table 1 shows the characteristics of the participants (*N* = 1510) divided into pregnant women with LBP (*n* = 822) and pregnant women without LBP (*n* = 688). No significant differences were found between the groups of pregnant women with and without LBP in terms of age, height, BMI, and weight.

Back pain was reported by 36.5% of the subjects, with an increase in the percentage to 54.4% during pregnancy. The factors associated with the increase in pain intensity among pregnant women with LBP were lying/sleeping (49.6%), sitting (38.7%), and walking (37.2%) followed by bending (32.8%), standing (24.1%), lifting heavy objects (21.9%), and physical activity (15.3%). The pain usually had a gradual onset (70.8%), but in some women (23.4%), it was difficult to determine the cause of the problem, whereas a small number of women experienced an abrupt onset. Women defined pain as temporary (44.9%), sharp (19.1%), radiating (18.4%), and persistent (17.6%). It was most commonly experienced during the day (62.0%), and after physical effort (48.2%), followed by at nighttime (39.4%), and was least severe in the morning (10.2%).

The mean pain intensity score in women with LBP was measured at 5.59 ± 1.72 on the VAS, and the pain onset was assessed as occurring at an average gestational age of 26.8 ±7.6 weeks. The mean disability score in pregnant women with pain was 11.6 ± 7.43 as measured by the ODI, whereas the impact the back problem had on the pregnant woman’s daily functional activities was 8.2 ± 4.34 according to the RMDQ measurement. Most women with LBP experienced minimal disability (55.5%) according to the ODI score, and mild disability (59.1%) according to the RMDQ. A significant correlation (Pearson’s correlation coefficient) was observed between the intensity of pain and the degree of disability (ODI; *p* < 0.001) as well as the effect pain had on the functional capacity of the pregnant women (RMDQ; *p* < 0.001). The occurrence of LBP has a strong correlation with LBP in previous pregnancies (*p* = 0.001). Those women who experienced period back pain before pregnancy were less likely to develop LBP (*p* < 0.001). Younger women were more likely to develop LBP (*p* < 0.001). This may be associated with lower education levels, which is also an LBP risk factor. Previous pregnancy childbirth type did not have a significant influence on the occurrence of LBP (*p* = 0.292). Physical activity before and during pregnancy was found to correlate significantly with a lower occurrence of LBP complaints among pregnant women (*p* < 0.001) (Table 2). 

## 4. Discussion

Lumbosacral pain in pregnant women is a common but still not entirely understood complaint [13,27,28]. It is considered to be a typical pregnancy-related discomfort since at least 50% of pregnant women experience the presence of back pain of varying intensity during pregnancy [6,29,30]. Researchers involved in the study of the subject have observed the complaint in 20–90% of pregnant women [9,12,28]. The epidemiological data are inconsistent in this respect, which may result from inaccuracies in the definition and classification of low back pain. Furthermore, many pain-intensifying factors, the subjectivity of assessment, and a variety of pain components (intensity, frequency of occurrence of pain, functional limitation, and well-being) affect the definitive evaluation of pain and discrepancies between the results of different studies [31,32]. Some of these complaints subside spontaneously, whereas some may evolve into chronic pain. This constitutes a major health concern. In his report, Vadivelu observed the problem of postpartum period misdiagnosis in women who keep suffering from pain in the lumbosacral spine radiating down the lower extremities to the knee, which is later reflected in the medical approach [33]. Sabino and Grauer note that back pain radiating down the leg to the knee in pregnancy and during the postpartum period is symptomatic of LBP in pregnant women. The authors argue that it is caused by changes taking place in the pelvic area (relaxation of pelvic ligaments), while sciatic pain also manifests itself in distal parts of the lower limbs [13]. Mogren and Pohjanen state that such pain usually occurs between 20 and 28 weeks of pregnancy, with a mean gestational age estimated at 22.1 weeks [11]. Our research showed that the onset of pain occurred at 26.8 ± 7.6 weeks, and it measured 5.59 ± 1.72 on the VAS. The onset of pain in a study conducted by Katonis et al. typically occurred at 27 weeks, which he supports by reporting 20–28 weeks as the first period during which pain occurs. However, according to Mogren and Pohjanen, the mean gestation age at the start of pain is in 22 weeks [12,34].

Body mass scores indicate that they may influence lumbar pain in pregnant women. One of the most significant changes that affects the musculoskeletal system is the increase in body mass during the gestational period. In the present study, the average BMI in women reporting pregestational low back pain was 22.72, which increased to 27.8 during pregnancy. The BMI values for women without LBP before pregnancy were 21.90 and 26.83, respectively. A similar correlation was reported by Mogren and Pohjanen in their study, which states a BMI of patients with pain at 24.57 before and 30.10 during pregnancy, while the scores for women without pain were 23.30 and 28.56, respectively [12]. Other researchers also identify the significance of body mass, particularly its substantial increase, as a risk factor associated with back pain during pregnancy [12,35]. However, some reports do not find any correspondence between the pregnant woman’s body weight and back pain [11,36].

Many researchers prove that if during the first pregnancy the woman experienced low back pain, she will experience recurrent episodes of pain in subsequent pregnancies [27,37]. Mota et al. [38] observed that in a group of pregnant women with LBP, 53.2% of them reported similar complaints in previous pregnancies. In our research, the group was considerably larger, as many as 85.5% of the women in the study had suffered from LBP in previous pregnancies. In their study, Wu et al. [27] listed strenuous work and lower back pain occurring both during the pregestational period and in previous pregnancies as risk factors associated with PGP and LBP. They suggested that these factors cause tissue damage, which later predisposes patients to developing symptoms. Some researchers acknowledge no correlation between the number of pregnancies and the occurrence of low back pain [35]. They do claim, however, that younger women [11,37,38], as well as those with lower levels of education [39], are more likely to experience LBP, which is consistent with the findings of our research (*p* < 0.001). In the first weeks following delivery, spontaneous improvement occurs and the low back pain decreases. For example, in a large randomised study, the pain resolved within the first 12 weeks postpartum in 99% of women, regardless of treatment [6]. However, when low back pain persists for 3–6 months postpartum, it is unlikely that it will settle spontaneously [40].

Regular pre-pregnancy physical activity reduces the occurrence of LBP during the gestational period [41,42], while strenuous work enhances the risk of LBP during pregnancy [31,41,42].

Physical activity during pregnancy significantly decreases low back pain [43,44], which agrees with the authors’ findings. The beneficial effect of exercise is similar to that observed generally across the population [45]. Doing stabilising exercises is of benefit to pregnant women suffering from low back and pelvic pain. What is more, physical activity during the gestational period may prevent future episodes of low back and pelvic pain from happening in subsequent pregnancies [46]. 

There also exists evidence suggesting that physical exercise during pregnancy (any kind of exercise land or water-based) may decrease pregnancy-related low back pain and that any form of exercise improves functional disability, as well as reducing sick leave more than antenatal care. Evidence from isolated studies suggests that acupuncture or craniosacral therapy improve pregnancy-related pelvic pain, while osteopathic therapy or multimodal intervention (manual therapy, exercise, and education) may also be beneficial [5].

The scope of the exercise programme and its duration must be individually tailored to suit the woman’s ability and health condition, as well as her enthusiasm and involvement. There are also forms of activity that are less popular but worth recommendation and considered safe for pregnant women, such as yoga, pilates, and aqua fitness. Introducing those forms of physical activity to birth centres would be highly beneficial. Doing specific fitness, breathing, and relaxing exercises holds many benefits for both mother and child. Some research shows that labour among physically active mothers is shorter (by 2–4 h on average), that the uterus functions better throughout the labour, that it is more likely for the child to arrive closer to the estimated due date, that there is a lower rate of episiotomy, that the risk of operative births is reduced, that blood loss during placental expulsion is decreased, that fewer breastfeeding problems are likely to occur, and that the time to full recovery postpartum is shorter. 

The benefits of the mother’s physical activity for the baby including the following: a lower incidence of the birth trauma, a facilitation of passing through the birth canal, better oxygenation during delivery, and a higher APGAR (Appearance, Pulse, Grimace, Activity, and Respiration) score. Regular physical activity benefits pregnancy, delivery and confinement, as well as decreasing perinatal complications [47,48,49,50].

Fast et al. [10] observed that about a third of pregnant women in the study experienced worsening of pain as the day progressed, whereas another third suffered from intensified pain at night-time, which interrupted sleep. The pain usually increased during standing, sitting, bending forward, lifting, and walking. Morino et al. [51] analysed daily activities inducing LBP. At all evaluations, movements—particularly sitting, standing up from a chair, and tossing and turning—were thought to be related to LBP.

The Oswestry Disability Index mean score among women reporting pain in our study was 11.6 ± 7.43 (the scores were between 0% to 76%). A strong correlation was observed between the ODI scores and pain intensity evaluated by the VAS (*p* < 0.001). In their studies Mohseni-Bandpei et al. [36] estimated the mean at 34.1% ± 15.8% (scores between 2% and 84%), and this also supports a significant correlation between the Oswestry Disability Index questionnaire score and the pain scale as measured by the VAS (*p* < 0.001). Apart from the aforementioned, no other relevant study has been found to document low back pain in pregnant women using the ODI questionnaire. Its scarce use in studies of the subject matter confirms that. The average intensity of low back pain among pregnant women, as measured by the Visual Analogue Scale in the authors’ study, was 5.72 ± 1.71. Wang et al (2004) estimated it at 4.56 ± 2.6, whereas Mohseni-Bandpei et al. [36] measured it at 5.1 ± 2.1. The results of this study and those of the adduced researchers are a proof of moderate low back pain intensity.

The problem is important in that there are no defined treatment strategies for pregnant women with LBP, which means that many of them are left untreated. Another drawback is pain persisting postpartum.

Despite the popularisation of a healthy and active lifestyle, the Polish people still tend to spend their leisure time passively. Women must abandon stereotypical attitudes and look for types of activities best suited to them. Many factors affect a woman’s condition during pregnancy, and it is necessary to choose the most optimal ones associated with a diet as well as a suitable physical activity.

## 5. Conclusions

Low back pain is a common and difficult problem for pregnant women. It was observed that a considerable number of pregnant women complains about the pain every day, while before the pregnancy, it is was an occasional occurrence. The predisposing factors for LBP are LBP in previous pregnancies, back pain during menstruation, a younger age, and a lack of physical activity. Most women with LBP experienced minimal disability according to the ODI score and mild disability according to the RMDQ.

This shows the scale of the problem and proves that it is important to find ways to reduce it. It may appear essential to provide education and awareness campaigns about possible treatments for low back pain.

## Figures and Tables

**Table 1:** Participant characteristics.

Variable	All Pregnant Women within the Group *N* = 1510	Pregnant Women with LBP *n* = 822	Pregnant Women without LBP *n* = 688
	*mean ± sd*	*mean ± sd*	*mean ± sd*
	*med; min–max*	*med; min–max*	*med; min–max*
**Age (years)**	28.6 ± 4.4	28 ± 4.3	29.3 ± 4.5
	28; 17–40	28; 18–40	29; 17–23
**Height (cm)**	166.3 ± 5.5	166.2 ± 5.6	166.4 ± 5.4
	165; 150–183	165; 150–180	165; 156–183
**Pregestational weight (kg)**	61 ± 11.1	62.9 ± 10.6	60.7 ± 9.7
	60; 40–99	60; 44–99	59; 40–90
**Gestational weight (kg)**	75.8 ± 11.1	77.3 ± 10.9	74.3 ± 11.3
	74; 51–115	76; 54–115	72; 51–111
**Pregestational BMI**	22.5 ± 3.6	22.7 ± 3.7	21.9 ± 3.2
	21.9; 15.4–33.7	22.3; 16.8–33.7	21.5; 15.4–33.2
**Gestational BMI**	27.4 ± 3.8	28.0 ± 3.6	26.8 ± 3.9
	27.2; 17.5–38,9	27.5; 20.1–38.6	26.8; 17.5–38.9
**Week of pregnancy**	37.6 ± 5.0	37.8 ± 4.6	37.3 ± 5.4
	39; 9–42	39; 9–42	39; 11–41
**Number of pregnancies**	1.6 ± 0.9	1.6 ± 0.8	1.6 ± 0.9
	1; 1–5	1; 1–5	1; 1–5

**Legend:** LBP–Low Back Pain; *sd*–standard deviation; *med*–median; *min*–minimum; *max*–maximum.

**Table 2:** LBP risk factors in pregnant women.

Variable	Frequency (%) Total *N* = 1510	Frequency (%) Women with LBP *n* = 822	Frequency (%) Women without LBP *n* = 688	Predicted Probability of LBP according to Analysed Factors
**Age (years)**	28.61 ± 4.39 *mean ± sd*	27.95 ± 4.29 *mean ± sd*	29.27 ± 4.53 *mean ± sd*	T (1431) = 5.77; *p* < 0.001
• ≤20	84 (5.6)	60 (7.3)	24 (3.5)	
• 21–25	218 (14.4)	138 (16.8)	80 (11.6)	OR = 1.06 (1.06–1.08)
• 26–30	720 (47.7)	408 (49.6)	312 (45.3)	
• 31–35	408 (27.0)	192 (23.4)	216 (31.4)	
• >35	80 (5.3)	24 (2.9)	56 (8.1)	
**LBP before pregnancy**				
• Yes	551 (36.5)	438 (53.3)	113 (16.4)	chi2(1) = 53.89; *p* < 0.001
• No	959 (63.5)	384 (46.7)	575 (83.6)	
**LBP during previous pregnancies**				
• Occurred	322 (21.3)	282 (34.3)	40 (5.8)	chi2(1) = 312.7; *p* = 0.001
• Did not occur	296 (19.6)	48 (5.8)	248 (36.0)	
• Not applicable (women in first pregnancies during the study)	892 (59.1)	492 (59.9)	400 (48.1)	
**Previous pregnancy childbirth type**				
• Natural birth	404	228 (15.1)	176 (11.7)	chi2(1) = 1.11; *p* = 0.292
• Caesarean section	198	102 (6.8)	96 (6.4)	
**Physical activity prior to pregnancy**				
• Yes	782 (51.8)	294 (35.8)	488 (70.9)	chi2(1) = 184.1; *p* < 0.001
• No	728 (48.2)	528 (64.2)	200 (29.1)	
**Physical activity during pregnancy**				
• Yes	742 (49.1)	246 (29.9)	496 (72.1)	chi2(1) = 254.8; *p* < 0.001
• No	768 (50.9)	576 (70.1)	192 (27.9)	
**Pregestational period back pain**				
• Yes	650 (43.0)	426 (51.8)	224 (36.7)	chi2(1) = 55.92; *p* < 0.001
• No	860 (57.0)	396 (48.2)	464 (67.4)	
**Education**				
• Primary	68 (4.5)	60 (7.3)	8 (1.2)	chi2(2) = 44.07; *p* < 0.001
• Secondary/Vocational	712 (47.2)	408 (49.6)	304 (44.2)	OR = 0.18 (0.08–0.36)
• University	730 (48.3)	354 (43.1)	376 (54.7)	OR = 0.13 (0.05–0.25)
**Occupational category**				
• Manual	476 (31.5)	176 (25.6)	300 (36.5)	OR = 2,17 (1.61–2.92)
• Non-manual (“white-collar”)	748 (49.6)	352 (51.2)	396 (48.2)	OR = 1,43 (1.09–1.88)
• Unemployed	286 (18.9)	160 (23.3)	126 (15.3)	chi2(2) = 27.26; *p* < 0.001

**Legend:** LBP–Low Back Pain; *sd —* standard deviation.
